# Perinatal mortality following assisted reproductive technology treatment in Australia and New Zealand, a public health approach for international reporting of perinatal mortality

**DOI:** 10.1186/1471-2393-13-177

**Published:** 2013-09-18

**Authors:** Elizabeth A Sullivan, Yueping A Wang, Robert J Norman, Georgina M Chambers, Abrar Ahmad Chughtai, Cynthia M Farquhar

**Affiliations:** 1 National Perinatal Epidemiology and Statistics Unit, The University of New South Wales, Sydney 2052 NSW, Australia; 2 Robinson Institute, School of Paediatrics and Reproductive Health, University of Adelaide, Adelaide 5005 SA, Australia; 3 Department of Obstetrics and Gynaecology, University of Auckland, Auckland 1020, New Zealand; 4 National Perinatal Epidemiology and Statistics Unit, Level 2, McNevin Dickson Building, Randwick Hospitals Campus, Randwick 2031, NSW, Australia

**Keywords:** Perinatal mortality, Assisted reproductive technology treatment, Embryo transfer, Stillbirth, Neonatal mortality

## Abstract

**Background:**

There is a need to have uniformed reporting of perinatal mortality for births following assisted reproductive technology (ART) treatment to enable international comparison and benchmarking of ART practice.

**Methods:**

The Australian and New Zealand Assisted Reproduction Database was used in this study. Births of ≥ 20 weeks gestation and/or ≥ 400 grams of birth weight following embryos transfer cycles in Australia and New Zealand during the period 2004 to 2008 were included. Differences in the mortality rates by different perinatal periods from a gestational age cutoff of ≥ 20, ≥ 22, ≥ 24, or ≥ 28 weeks (wks) to a neonatal period cutoff of either < 7 or < 28 days after birth were assessed. Crude and specific (number of embryos transferred and plurality) rates of perinatal mortality were calculated for selected gestational and neonatal periods.

**Results:**

When the perinatal period is defined as ≥ 20 wks gestation to < 28 days after birth, the perinatal mortality rate (PMR) was 16.1 per 1000 births (n = 630). A progressive contraction of the gestational age groups resulted in marked reductions in the PMR for deaths at < 28 days (22 wks 11.0; 24 wks 7.7; 28 wks 5.6); and similarly for deaths at < 7 days (20 wks 15.6, 22 wks 10.5; 24 wks 7.3; 28 wks 5.3). In contrast, a contraction of the perinatal period from < 28 to < 7 days after birth only marginally reduced the PMR from 16.2 to 15.6 per 1000 births which was consistent across all gestational ages.

The PMR for single embryo transfer (SET) births (≥ 20 weeks gestation to < 7 days post-birth) was significantly lower (12.8 per 1000 SET births) compared to double embryo transfer (DET) births (PMR 18.3 per 1000 DET births; p < 0.001, Fisher’s Exact Test). Similarly, the PMR for SET births (≥ 22 weeks gestation to < 7 days post-birth) was significantly lower (8.8 per 1000 SET births, p < 0.001, Fisher’s Exact Test) when compared to DET births (12.2 per 1000 DET births). The highest PMR (50.5 per 1000 SET births, 95% CI 36.5-64.5) was for twins following SET births (≥ 20 weeks gestation to < 7 days post-birth) compared to twins following DET (23.9 per 1000 DET births, 95% CI 20.8-27.1).

**Conclusion:**

Reporting of perinatal mortality of ART births is an essential component of quality ART practice. This should include measures that monitor the impact on perinatal mortality of multiple embryo transfer. We recommend that reporting of perinatal deaths following ART treatment, should be stratified for three gestation-specific perinatal periods of ≥ 20, ≥ 22 and ≥ 28 completed weeks to < 7 days post-birth; and include plurality specific rates by SET and DET. This would provide a valuable international evidence-base of PMR for use in evaluating ART policy, practice and new research.

## Background

There is international agreement of the importance of perinatal mortality measures for monitoring pregnancy and childbirth with routine reporting at a country and regional level [1]. This monitoring does not commonly extend to births following assisted reproductive technology (ART) treatment with only Australia and New Zealand, Latin America, Japan and Canada including perinatal mortality measures in their most recent ART reports [2-5]. In contrast, the ART reports from the United States and Europe present data on the births of almost 150,000 babies but no information on mortality [6,7]. The lack of perinatal mortality data is a major health information gap in benchmarking the quality of ART policy and the safety of practice.

Internationally, monitoring and comparison of perinatal mortality is limited by the wide variation in the definitions of birth, stillbirth and perinatal death used in countries that practice ART (Table 1) [3,4,8,9]. The World Health Organization (WHO) recommends for international comparison that the definition of births be restricted to 1000 grams and/or 28 weeks gestation for all perinatal measures [10] but the usefulness of this definition for monitoring perinatal mortality is limited for births following ART where there are proportionately high rates of multiple birth and preterm birth [11]. In 2009, the International Committee Monitoring Assisted Reproductive Technologies (ICMART) and the WHO sought to limit the perinatal period for birth following ART treatment to ≥20 completed weeks of gestational age to <7 completed days after birth regardless of birth weight [12]. However, for many countries birth data are not available for gestations of <22 weeks limiting the utility of international comparison based on the ICMART/WHO definition of birth.

**Table 1:** Definitions of perinatal mortality internationally

**Country or Institution**	**Perinatal death**		
	**Fetal death**		**Neonatal death**
	**Gestational age weeks**	**Birthweight grams**	**Days**
New Zealand	20	400	<28
International Committee Monitoring Assisted Reproductive Technologies (ICMART)	20	–	< 7
Australia	20	400	< 28
Canada	20	–	< 7
United States	20	350	< 28
Latin America	20	–	< 28
Europe^1^	22	500	< 7
Europe^2^	22	–	< 7
Europe^3^	22	1000	< 7
Japan	22	–	< 7
World Health Organization (WHO) (national reporting)	22	500	< 7
Europe^4^	24	–	< 28
UK	24		<7
WHO (international comparison)	28	1000	< 7
Europe^5^	28	–	< 7

^1^Includes Norway, France, Finland, Estonia, Belgium, Netherlands, Malta.

^2^Includes Spain (Valencia), Latvia, Lithuania, Denmark, Slovak Republic.

^3^Includes Czech Republic.

^4^Includes Ireland, Hungary, Portugal.

^5^Includes Sweden, Greece.

Further, when agreeing on the perinatal measures to monitor ART, the impact of ART treatment practices needs to be considered, most notably the number of embryos transferred. The literature show increased risk of multiple gestation with the transfers of two or more embryos [13]. For example in 2010, in Quebec, Canada, the change in policy to elective single embryo transfer (SET) (from 1.6% of cycles to 50%) resulted in a reduction in the multiple pregnancy rate from 25.6% in 2009 to 3.7% in the first three months of implementation of a policy of SET [14].

Reducing the number of embryos per transfer is essential to lower both perinatal morbidity and mortality for ART births [13,15]. Among the risk factors for perinatal mortality, iatrogenic multiple gestation pregnancy can be considered the most significant preventable risk for women undergoing ART treatment. Research studies and subsequent policy implementation of SET in New Zealand, Sweden, Belgium and Quebec have reduced the proportion of double embryo transfers (DET) and multiple gestations but have not routinely reported embryo-transfer specific perinatal mortality [13,16-18]. This lack of reporting of embryo-transfer specific rates of perinatal mortality limits evaluation of the policy of SET [19]. The aim of this study is to develop an approach for standardization of reporting of PMR following ART treatment for international comparison. We applied commonly used American and European perinatal periods to the Australian and New Zealand ART registry data, to investigate the changes in PMRs stratified by plurality and number of embryos transferred.

## Methods

A review of reporting perinatal mortality of ART births was conducted. The 2004-2008 data from the Australia and New Zealand Assisted Reproductive Database (ANZARD) were used to assess the differences in the definitions of perinatal periods based on internationally used gestational age at birth and number of days after birth. ANZARD is a census of all initiated ART treatment cycles undertaken in Australia and New Zealand. Items on ANZARD are collated annually by the National Perinatal Epidemiology and Statistics Unit (NPESU), in a de-identified format, from all fertility centres within Australia and New Zealand. Fertility centres as part of their quality assurance are required to report ART treatment and outcomes to ANZARD. Birth outcomes collected in ANZARD include birth status, gestational age, birth weight, date of neonatal death and congenital anomalies. The fertility clinic data that makes up ANZARD is audited for completeness and accuracy on an annual basis as part of accreditation for fertility clinics through the Reproductive Technology Accreditation Committee.

### Australian definition of birth and the perinatal period

The Australian definition of a birth is all live and stillbirths (fetal deaths) of ≥20 weeks gestation and/or ≥400 grams of birth weight. The perinatal period is defined for liveborns to <28 complete days after birth. The study population includes all births following ART treatments that occurred during the period 1 January 2004-31 December 2008. A total of 50,258 ART births were used to calculate the PMR according to different international published definitions.

### Comparison with international definition

The inclusion criteria for fetal deaths varied internationally by gestation and or birthweight for both ART and spontaneous births (Table 1). The gestational age low limits for fetal death vary between countries, ranging from 16 to 28 weeks [20]. In Australia, New Zealand, the United States, Canada and Latin America, a gestational age of ≥20 complete weeks is used to define a birth [3,4,8,21-25]. In contrast, there is marked variability in the definition of birth among European countries. For some countries (e.g. Norway, France and Finland) the inclusion criteria for a birth is a gestational age of ≥22 complete weeks [26-28], while for others the gestational age is either ≥24 (United Kingdom) [29] or ≥28 weeks (Sweden and Denmark) [28,30]. Elsewhere, birthweight is used to define a birth [23,28,31]. In other countries, birthweight is not one of the inclusion criteria [3,4]. The definitions of neonatal period also vary by countries and regions using either <7 completed days [29] or <28 completed days after birth [24,25] (Table 1).

### Definition of stillbirth, neonatal death and perinatal death

Perinatal mortality rates for ART births were abstracted from published studies or reports. Studies/reports needed to satisfy the following criteria to be included: [1] data extracted from a national or regional ART registry; [2] overlapping data for the time period of the study (2004-2008); and [3] mortality measures with clear definitions. There were no published reports from the United Kingdom on perinatal mortality so the statistics were calculated using publicly available data [23,31]. The perinatal periods were defined in various ways dependent upon the country, region or international organization (Table 1).

Table 2 details the definitions of fetal, neonatal and perinatal mortality used in this study. Eight commonly used definitions of perinatal periods (Tables 1, 2) were examined to assess the differences in PMRs. To demonstrate the importance of direct comparison of PMRs for ART births using the same perinatal period, the definitions of perinatal mortality which were published in journal articles and national/regional reports of Canada, the United Kingdom, Sweden, Japan, Finland, Norway and Latin America were adopted to compute specific PMRs for Australia and New Zealand.

**Table 2:** Variations in the definitions of fetal, neonatal and perinatal death

**Gestational age at birth dependent on country or organisation definition (weeks)**				**Survival after birth (days)**	
≥ 20	≥ 22	≥ 24	≥ 28	< 7	< 28
**Fetal death**				**Neonatal death**	
Death prior to complete expulsion or extraction from its mother of a product of conception at a defined number of completed weeks of gestational age				Death that occurs of a liveborn infant in the neonatal period defined as either <7 or <28 completed days after birth	
**Perinatal death**					
Any fetal and/or neonatal death that occurs within the defined perinatal period					

### Statistical analysis

The rates of fetal, neonatal and perinatal mortality were expressed per 1000 births. Fetal and PMRs were calculated using all births (live births and fetal deaths) as the denominator. Neonatal mortality rates were calculated using live births as the denominator. According to the literature, PMRs for the periods listed in Table 2 were computed. The differences in PMRs at different gestational ages (20, 22, 24 and 28 weeks) and different number of days after birth (<7 or <28 days) were compared. These were in accordance with the countries’ or organizations’ PMR definitions. Plurality (singletons and twins) and number of embryos transferred (single and double) were used for stratification. The difference in PMRs of SET and DET births was tested by Fisher’s exact method. Data were analyzed using SPSS, version 19.0 (SPSS Inc., Chicago, Ill, USA).

### Ethics

Ethics approval for this study was granted by the Human Research Ethics Advisory Panel of the University of New South Wales, Australia (Reference 2010-7-55). Approval for use of these data in research was received from the Fertility Society of Australia and New Zealand.

## Results

### Rates of perinatal mortality for Australia and New Zealand using different international perinatal period scopes

Table 3 presents the rates of fetal, neonatal and perinatal mortality following ART in Australia and New Zealand by applying the four most common gestational age criteria and either <7 and <28 completed days after a live birth. The inclusion of earlier gestational ages resulted in increased rates of fetal, neonatal, and perinatal mortality. When the perinatal period was defined as ≥ 28 weeks’ gestation to < 7 days after birth, (WHO definition) the PMR was 5.3 per 1000 births, this almost tripled to 16.1 per 1000 births when the (Australian definition) ≥ 20 weeks gestational age to < 28 days after birth was applied. Over 64% of fetal deaths (n = 402) were excluded when using the WHO definition as compared to the Australian definition. Similarly, almost 80% of neonatal deaths (n = 143) were not counted when changing the scope of perinatal period used in Australia to that used by WHO. The majority of neonatal deaths occurred < 7 days after birth, with 86% of neonatal deaths for babies born at a gestational age ≥ 20 weeks being early neonatal deaths compared to 74% for gestational age ≥ 28 weeks.

**Table 3:** Australian and New Zealand perinatal mortality following ART treatment by different international definitions of perinatal period

**Perinatal period**		**Total births**	**Fetal deaths**		**Neonatal deaths**		**Perinatal mortality rate**
**From: gestational age weeks**	**To: days survived after birth**	**Number**	**Number**	**Cumulative percent**	**Number**	**Cumulative percent**	**Per 1000 births**
≥ 20^1^	< 28	50252	625	100.0	182	100.0	16.1
≥ 22	< 28	49981	416	66.6	135	74.2	11.0
≥ 24	< 28	49760	289	46.2	95	52.2	7.7
≥ 28	< 28	49212	223	35.7	53	29.1	5.6
≥ 20^2^	< 7	50252	625	100.0	157	100.0	15.6
≥ 22	< 7	49981	416	66.6	110	70.1	10.5
≥ 24	< 7	49760	289	46.2	73	46.5	7.3
≥ 28^3^	< 7	49212	223	35.7	39	24.8	5.3

^1^Australian/New Zealand definition includes all live and stillbirths of ≥20 completed weeks of gestation and/or ≥400 grams of birthweight and death of a neonate <28 complete days after birth.

^2^ICMART defines the perinatal period as births of ≥20 completed weeks of gestation to <7 completed days after birth regardless of birthweight.

^3^WHO defines the perinatal period births of ≥28 completed weeks of gestation to <7 completed days after birth regardless of birthweight.

### Comparative international rates of perinatal related mortality measures

Table 4 presents the computed PMRs for Australia and New Zealand using the same scope and definition of other countries and regions. The crude PMR was significantly lower in Australia and New Zealand as compared to Canada, the United Kingdom, Japan and Latin America. In contrast, the singleton specific PMR was significantly higher in Australia and New Zealand compared to Sweden, Finland and Latin America but significantly lower than in Japan. These crude rates have not been adjusted for the number of embryos transferred or other maternal and treatment factors but show the enormous variability in perinatal mortality rates following ART treatment across countries and regions.

**Table 4:** **Comparison of international perinatal mortality definitions and rates as applied to Australia and New Zealand**^**1**^

	**Perinatal period**		**Perinatal mortality rate**			
**Country/region, year, source of data**	**From: gestational age weeks**	**To: days survived after birth**	**Plurality or treatment factor**	**Published**	**Computed Australia and New Zealand**^**1,2,3**^	**P value**
Canada, 2007	20	28	Singletons	12.6	12.4	0.93
			Twins	31.8	27.4	0.28
Gunby et al. 2011						
			HOM^4^	55.6	101.2	0.18
			Overall	21.8	16.1	<0.01
United Kingdom, 1991-2010	24	28	Overall	17.8	7.7	<0.01
HFEA, 2011^5^						
Sweden, 2002-2006	28	7	Singletons	3.7	5.6	0.03
Sazonova et al. 2011						
Japan, 2006	22	7	Singletons	16.3	8.3	<0.01
Fujii et al. 2010						
Finland, 1995-2006	22	7	FET^6^ singletons	4.4	6.0	0.38
Pelkonen et al. 2010						
			Fresh singletons	5.4	6.4	0.52
Latin America, 1990-2009	20	28	Singletons	9.9	12.1	<0.01
			Twins	27.8	26.2	0.41
Zegers Hochschild et al. 2011						
			HOM^4^	77.5	98.2	0.16
			Overall	23.7	15.6	<0.01
Norway, 1984-2006	22	7	Singletons	9.5	8.3	0.27
Romundstad et al. 2008						

^1^Calculated from ANZARD data, 2004-2008.

^2^Calculated using the same time period for the years of data 2004–2008; if the data includes the 5 years of data plus additional data only 2004-2008 Australian and New Zealand data used.

^3^Differences in the computed Australian and New Zealand rates reflect the different time periods.

^4^HOM higher order multiple excludes twins.

^5^Calculated from publically available HFEA data.

^6^FET frozen embryo transfer.

### Benchmarking of perinatal mortality after ART

Table 5 shows the perinatal mortality for births for two perinatal periods stratified by the number of embryos transferred and plurality. The PMR for SET births (from ≥20 weeks gestation to <7 days after birth) was significantly lower (12.8 per 1000 SET births) compared to DET births (PMR 18.3 per 1000 DET births; p < 0.001, Fisher’s Exact Test). Similarly, the PMR for SET births (from ≥22 weeks gestation to <7 days after birth) was significantly lower (8.8 per 1000 SET births, p < 0.001, Fisher’s Exact Test) when compared to DET births (12.2 per 1000 DET births). Thirty-two percent of perinatal deaths of singletons and twins were excluded when the two week narrower perinatal period of ≥22 weeks gestation to <7 days after birth was used. The highest rate of fetal mortality is for twins following SET, there was a non significant fall in the fetal death rate when the narrower perinatal period of ≥22 weeks gestation was used.

**Table 5:** Fetal, neonatal and perinatal mortality rates of suggested perinatal period by single and double embryo transfers

**Number of embryos transferred and plurality**	**Number**			**Mortality rate and (95% CI) /denominator**		
	**Total births**	**Fetal deaths**	**Neonatal deaths**	**Fetal 1000 births**	**Neonatal /live births**	**Perinatal /1000 births**
**From ≥ 20 weeks gestational age to < 7 survival days after birth**						
*Single embryo transfer*						
Singletons	24199	235	38	9.7(8.5-11.0)	1.6(1.1-2.1)	11.3(9.9-12.6)
Twins	990	41	9	41.4(28.7-54.1)	9.5(3.3-15.7)	50.5(36.5-64.5)
All births^1^	25220	277	47	11.0(9.7-12.3)	1.9(1.3-2.4)	12.8(11.4-14.2)
*Double embryo transfer*						
Singletons	15115	172	29	11.4(9.7-13.1)	1.9(1.2-2.6)	13.3(11.5-15.1)
Twins	9150	151	68	16.5(13.9-19.1)	7.6(5.8-9.4)	23.9(20.8-27.1)
All births^1^	24525	343	106	14.0(12.5-15.5)	4.4(3.5-5.2)	18.3(16.6-20.0)
*All transfers*^*2*^						
Singletons	39624	410	69	10.3(9.3-11.3)	1.8(1.3-2.2)	12.1(11.0-13.2)
Twins	10292	193	77	18.8(16.1-21.4)	7.6(5.9-9.3)	26.2(23.1-29.4)
All births^1^	50252	625	157	12.4(11.5-13.4)	3.2(2.7-3.7)	15.6(14.5-16.7)
**From ≥ 22 weeks gestational age to < 7 survival days after birth**						
*Single embryo transfer*						
Singletons	24200	159	28	6.6(5.5-7.6)	1.2(0.7-1.6)	7.7(6.6-8.8)
Twins	990	28	6	28.3(17.8-38.8)	6.2(1.2-11.2)	34.3(22.8-45.9)
All births^1^	25108	187	34	7.4(6.4-8.5)	1.4(0.9-1.8)	8.8(7.6-10.0)
*Double embryo transfer*						
Singletons	15046	118	17	7.8(6.4-9.3)	1.1(0.6-1.7)	9.0(7.5-10.5)
Twins	9075	95	50	10.5(8.4-12.6)	5.6(4.0-7.1)	16.0(13.4-18.6)
All births^1^	24353	225	72	9.2(8.0-10.4)	3.0(2.3-3.7)	12.2(10.8-13.6)
*All transfers*^*2*^						
Singletons	39461	279	47	7.1(6.2-7.9)	1.2(0.9-1.5)	8.3(7.4-9.2)
Twins	10199	124	56	12.2(10.0-14.3)	5.6(4.1-7.0)	17.6(15.1-20.2)
All births^1^	49981	416	110	8.3(7.5-9.1)	2.2(1.8-2.6)	10.5(9.6-11.4)

^1^include higher order multiples.

^2^include transfers of three or more embryos.

## Discussion

This study shows that the wide variation in the measures used for reporting perinatal mortality at a country and regional level limits direct comparison and evaluation of ART policy and best practice internationally. Monitoring of perinatal mortality is an essential component of any quality system that seeks to improve ART services and the related maternal and perinatal outcomes. Unlike natural conception, ART treatment involves elective decision making on the part of the clinician and couple, most notably about the number of embryos transferred. The number of embryos transferred is highly correlated with the risk of multiple gestation pregnancies, which remain prevalent in most countries [13,14]. Therefore, it is essential that routine perinatal mortality measures following ART are able to monitor the impact of embryo transfer practices among countries.

This study demonstrates the impact different definitions of perinatal mortality have on PMRs. The PMR for Australia and New Zealand was 16.1 per 1000 births (Australian definition ≥ 20 weeks gestation to <28 days post-birth) compared to 5.1 per 1000 births, when the more limited WHO recommended definition for international comparison (≥ 28 weeks gestation to <7 days post-birth) was applied [10,23]. Application of the WHO definition results in the exclusion of 1,040 (9.5%) preterm births, 402 (64.3%) stillbirths and 143 (78.6%) neonatal deaths. These differences can be overcome by presenting data for three gestational time periods (≥ 20 weeks, ≥ 22 weeks, ≥ 28 weeks and limiting the perinatal period to 7 days post birth) where the majority of neonatal deaths occur. This would allow international comparison. The first of these proposed definitions would be consistent with ICMART / WHO glossary definition of a perinatal death and could be adopted as a standard for international reporting [12]. The inclusion of ≥20 completed weeks’ gestation would allow greater utility to investigate the impact of ART and multiple gestation from the provision of neonatal services.

Most countries follow the WHO definition (gestational age of 22 weeks or birth weight of 500 grams and <7 days) for national reporting of perinatal mortality. However, some countries only use gestational age and others birth weight [30]. Birth weight is included in the definition of perinatal mortality, particularly in low income countries, as it is easier to measure and less prone to error than calculations of gestational age from the last menstrual period [32]. In contrast, Mohangoo et al. suggested that mortality rates are usually underestimated if the case definition includes birth weight rather than gestational age. This was demonstrated in the Euro-Peristat project which reported comparatively lower fetal death rates when birth weight criteria was used rather than gestational age [33]. Therefore, most of the high income countries prefer to use gestational age, given it is more sensitive predictor for the perinatal outcome [20]. In addition, gestational age of births following ART treatment are calculated based on date of embryo transfer and date of delivery which are usually collected in ART registers [34,35]. Regardless of which criteria are used, the variation in birth registration practices and in scope of perinatal periods significantly changes the international rankings of countries based on mortality rates [36]. Standardization in perinatal data collection and reporting will help in monitoring ART treatment practices and is consistent with the recommendations from the Global report on preterm birth and stillbirth [20].

The clinical relevance of benchmarking of ART practice with the reporting of embryo-specific- and plurality-specific-rates of perinatal mortality is evident. The transfer of two or more embryos significantly increases the risk of multiple gestation pregnancy [13,37].Multiple gestation pregnancies are at higher risk of fetal, neonatal and perinatal mortality than singleton pregnancies [15]. In this study, SET was associated with a significantly lower PMR for all births when compared to DET. Double embryo transfers were associated with one extra death per 200 births. In contrast, twin gestations following SET resulted in a significantly higher PMR compared to DET.

Further, there is wide variation in the number of embryos transferred by countries and regions and the policy framework for SET [19]. The international comparison of perinatal mortality is complicated by the variability in the scope and quality of death data between countries. This includes differing registration criteria and denominator populations and variability in data collection practices. The higher PMR for Australia and New Zealand compared to Sweden and Finland [27,38] is likely related to the earlier adoption and higher proportions of SET in Sweden and Finland compared to Australia and New Zealand. In contrast, the lower PMR for Australia and New Zealand when compared to Canada, the United Kingdom and Latin America likely reflects the higher proportion of SET in Australia and New Zealand than those countries and regions [3,4,31]. Additionally, studies have reported better perinatal outcomes of singletons following SET when compared to singletons following DET [39,40]. It has also been suggested that twins following SET (considering monozygotic twins) had a higher risk of perinatal death than twins following DET (considering dizygotic twins) [41,42]. Therefore, stratification by number of embryos transferred and plurality would allow making direct assessment of the risk of perinatal mortality due to DET compared to SET, multiples compared to singletons and presumptive monozygotic twins compared to dizygotic twins.

This study used five years of national level Australian and New Zealand data with approximately 10,000 births per year to minimize the effects of random variation and to maximize the stability and reliability of the mortality rates. There are some limitations of this study. First, with all registry studies there is the potential for information biases associated with differential ascertainment of fetal and neonatal deaths from births. However, a recent abstract reported a similar PMR for ART births to ANZARD using the national perinatal data collection [43]. Alternatively, if the quality or completeness of perinatal mortality data in ART registries is limited, data linkage among ART, birth and perinatal death registers is an option. This has the potential to enhance the validity of the death data internationally as well as providing information on cause of death [44]. Secondly, we did not stratify for other patient and treatment related factors even though published research has shown that ART is generally associated with more maternal and fetal complications, which vary according to the type of procedure and type of treatment [45-47]. Instead, we only included the most critical factors for perinatal mortality, namely the number of embryos transferred and plurality to present a public health approach for reporting perinatal mortality internationally.

## Conclusion

International benchmarking of perinatal mortality of ART births provides essential information to improve the safety and quality of ART practice. Due to the persisting high rates of multiple embryo transfers and of multiple birth internationally best practice is to report all mortality measures by number of embryo transfers (SET, DET and all transfers) and plurality (singletons, twins and all). The ICMART / WHO recommended a perinatal period of ≥ 20 completed weeks of gestational age to < 7 completed days after birth which would optimize the standardization of reporting fetal and neonatal deaths following ART treatment. Noting, this is currently not possible in Europe and many other countries. Reporting PMR specifically for three perinatal periods: ≥ 20 weeks gestational age to < 7 days after birth; ≥22 weeks gestational age to <7 days after birth and ≥ 28 weeks gestational age to <7 days after birth would allow international benchmarking of mortality of ART births. This would rapidly provide an international evidence-base to evaluate ART practice and policy against perinatal mortality.

## Abbreviations

ANZARD: Australia and New Zealand Assisted Reproductive Database (ANZARD); ART: Assisted reproductive technology; DET: Double embryo transfer; PMR: Perinatal mortality rate; SET: Single embryo transfer.

## Competing interests

All authors have no competing interests in relation to this work. We declare: no support from any organisations for the submitted work. There is a financial relationship between the University of New South Wales and the Fertility Society of Australia. The Fertility Society of Australia funds the National Perinatal Epidemiology and Statistics Unit to manage National Perinatal Epidemiology and Statistics Unit, University of New South Wales. The Fertility Society of Australia did not provide research funding for the study. Robert Norman has a financial interest in an IVF unit, Fertility SA. We declare: no other support from any organisations for the submitted work.

## Authors’ contributions

EAS led the study design, method investigation and preparing the manuscript. YAW was involved in study design, method investigation, data analysis and revising the manuscript. RJN, AAC and CMF were involved in the methods and revising the manuscript. All authors read and approved the final manuscript.

## Authors’ information

EA Sullivan, MBBS, MPH, MD. Professor and the director of National Perinatal Epidemiology and Statistics Unit, the University of New South Wales.

YA Wang, BMed, MPH, PhD. Biostatistician and Senior Lecturer of National Perinatal Epidemiology and Statistics Unit, the University of New South Wales.

RJ Norman, BSc (Hons), MB ChB (Hons), MD. Professor and the director of Robinson Institute, School of Paediatrics and Reproductive Health, University of Adelaide.

GM Chambers, BAppSci(MLS), MBA, PhD. Senior Lecturer of National Perinatal Epidemiology and Statistics Unit, the University of New South Wales.

AA Chughtai, (MBBS, MPH) Research officer of National Perinatal Epidemiology and Statistics Unit, the University of New South Wales.

CM Farquhar, MBBS, PhD. Professor Department of Obstetrics and Gynaecology and National Women’s Health, University of Auckland.

## Pre-publication history

The pre-publication history for this paper can be accessed here:

http://www.biomedcentral.com/1471-2393/13/177/prepub
